# Radiofrequency Catheter Ablation Improves the Quality of Life Measured with a Short Form-36 Questionnaire in Atrial Fibrillation Patients: A Systematic Review and Meta-Analysis

**DOI:** 10.1371/journal.pone.0163755

**Published:** 2016-09-28

**Authors:** Yun Gi Kim, Jaemin Shim, Jong-Il Choi, Young-Hoon Kim

**Affiliations:** From the Division of Cardiology, Department of Internal Medicine, Korea University Medical Center, Seoul, Korea; Kurume University School of Medicine, JAPAN

## Abstract

**Background:**

The main purpose of performing radiofrequency catheter ablation (RFCA) in atrial fibrillation (AF) patients is to improve the quality of life (QoL) and alleviate AF-related symptoms. We aimed to determine the qualitative and quantitative effects of RFCA on the QoL in AF patients.

**Methods:**

We performed a systemic review and meta-analysis using a random effects model. We searched for the studies that reported the physical component summary score (PCS) and mental component summary score (MCS) of the short form-36, a validated system to assess and quantify the QoL, before and after RFCA in AF patients. PCS and MCS are T-scores with a mean of 50 and standard deviation of 10.

**Results:**

Of the 470 studies identified through systematic search, we included 13 studies for pre-RFCA vs. the post-RFCA analysis and 5 studies for treatment success vs. AF recurrence analyses. In the pre-RFCA vs. post-RFCA analysis, RFCA was associated with a significant increase in both the PCS (weighted mean difference [WMD] = 6.33 [4.81–7.84]; *p* < 0.001) and MCS (WMD = 7.80 [6.15–9.44]; *p* < 0.001). The ΔPCS (post-RFCA PCS–pre-RFCA PCS) and ΔMCS values were used for the treatment success vs. AF recurrence analysis. Patients with successful ablation had a higher ΔPCS (WMD = 7.46 [4.44–10.49]; *p* < 0.001) and ΔMCS (WMD = 7.59 [4.94–10.24]; *p* < 0.001).

**Conclusions:**

RFCA is associated with a significant increase in the PCS and MCS in AF patients. Patients without AF recurrence after RFCA had a better improvement in the PCS and MCS than patients who had AF recurrence.

## Introduction

Antiarrhythmic drug therapy is considered as a first line treatment for the management of atrial fibrillation (AF). Antiarrhythmic drugs (AAD), however, do not reduce the adverse clinical outcomes in AF patients [1] and are associated with serious side effects [1–3]. The maintenance rate of sinus rhythm is also disappointing [1]. Since the 2000’s, electrical pulmonary vein isolation (PVI) through radiofrequency catheter ablation (RFCA) has emerged as a new treatment option for drug refractory AF [3–7]. Several trials have also studied the efficacy of RFCA in AAD naive patients [8–10]. Although these studies have demonstrated a superiority of RFCA in maintaining sinus rhythm in AF patients, current available trials indicate that both AAD and RFCA do not reduce the mortality or serious complications such as stroke [1, 6, 9–11]. Consequently, reducing AF-related symptoms and improving the quality of life (QoL) is the cornerstone of AF treatment [12, 13].

The QoL, at first glimpse, is rather abstract. However, through a systematic questionnaire and direct questioning, the QoL can be quantified [12–14]. Several questionnaires and scoring systems are available to evaluate the QoL in AF patients such as the SF-36, SF-12, EuroQoL, AF-QoL, EHRA AF symptom scale, and AFQLQ [14]. Among those scoring systems, the short form-36 (SF-36) is the most widely validated system and extensive studies have used the SF-36 to assess the QoL in AF patients [14]. The SF-36 scoring system is measured through a 36-item questionnaire and consists of eight components: general health, physical functioning, physical role, bodily pain, mental health, social functioning, emotional role, and vitality [15]. Standardized scores ranging from 0 (worst health) to 100 (best health) are provided for each component. The SF-36 scoring system also generates a physical component summary score (PCS) and mental component summary score (MCS) based on its eight components.

Based on the data from recent clinical trials, RFCA is likely to be associated with a significant improvement in both the PCS and MCS [4, 5, 9, 10]. However, only a few studies have studied the QoL as a primary end-point and the degree of the improvement in the PCS and MCS varies between study to study. Therefore, a pooled analysis of the available studies describing the relationship between RFCA and the changes in the PCS and MCS may provide critical and clinically useful information with respect to performing RFCA in AF patients. We performed a systematic review and meta-analysis of the studies to elucidate the effect of RFCA on the QoL in AF patients measured with SF-36. We also evaluated the importance of the treatment success (no AF recurrence after RFCA) on the QoL in AF patients undergoing RFCA.

## Methods

### Search strategy

A comprehensive systematic search was performed by two investigators (Y.G.K and J.M.S) using the Pubmed (www.pubmed.gov) and Scopus (www.scopus.com). US National Institutes of Health registry of clinical trials (www.clinicaltrials.gov) and conference proceedings from the American College of Cardiology, European Society of Cardiology, and American Heart Association were also checked. After performing the electronic search, a manual examination of the bibliographies of the included articles, review articles, meta-analyses, and editorials was conducted. The main key words used for the electronic search were *atrial fibrillation*, *AF*, *A-fib*, *ablation*, *SF-36*, *short form-36*, *and short form 36*. The exact search strategies are presented in S1 Table. Searches were not restricted by language, country, study period, study design, or sample size. The last search was performed in December 2015.

### Study selection

In order to select studies appropriate for the meta-analysis, two authors (Y.G.K and J.M.S) independently identified duplicates, reviewed titles and abstracts, and ruled out irrelevant articles. Subsequently, a full article review was conducted on the studies with possible inclusion to determine their eligibility. Disagreements between the authors were resolved by discussion.

Studies reporting the results of the PCS and MCS before and after RFCA in AF patients were included for the analysis. All types of AF (paroxysmal, persistent, long-standing, and permanent) were eligible for the analysis. Studies were excluded if they had only the pre-RFCA or post-RFCA scores of the SF-36. Studies evaluating the QoL in AF patients receiving surgical ablation, nodal ablation, AAD, or cryoablation were also excluded. In addition, studies regarding RFCA of atrial flutter complicating AF were not included.

### Data extraction and quality assessment

The summarized data of each published article were collected for the analysis. Data were extracted through a standardized form, which was in accordance with the Preferred Reporting Items for Systematic reviews and Meta-Analyses (PRISMA) statement [16]. The following items were gained from the individual studies included for the analysis: journal name, author name, publication year, study design, type of AF, percentage of each AF type, exact type of ablation, success rate of RFCA, complications related to RFCA, anticoagulation regimen, whether AF patients were refractory to prior AAD, demographic and clinical information of the study patients (mean age, sex, left atrial size, left ventricular ejection fraction, and duration of AF), pre-RFCA and post-RFCA SF-36 scores (PCS & MCS), and the time interval between the pre-RFCA and post-RFCA SF-36 scores. If the PCS and MCS of the treatment success and failure group were both available, the scores were collected separately in addition to the whole cohort results. The mean, standard deviation (SD), sample size, paired sample *p*-value (pre-RFCA vs. post-RFCA), and independent sample *p*-value (treatment success group vs. AF recurrence group) were collected for both the PCS and MCS. The Newcastle-Ottawa scale was utilized to assess the quality of eligible non-randomized observational studies. However, we did not exclude individual studies based on the results of the Newcastle-Ottawa scale.

### Statistical analysis

The primary analysis evaluated whether the post-RFCA PCS and MCS were better than the pre-RFCA scores. The pre-RFCA and post-RFCA scores are basically a paired sample, since it is drawn from the same patients, and we performed a meta-analysis comparing the paired data (pre and post mean scores). In order to calculate the effect size of the individual studies, either of two combinations of raw data were needed: (i) the means of the PCS or MCS (both pre-RFCA and post-RFCA), sample size, and a paired *p*-value; or (ii) the mean differences of the PCS or MCS (between pre-RFCA and post-RFCA), sample size, and a paired *p*-value. Studies that provided only the means, SD, and sample size were not able to calculate the effect size since a meta-analysis of paired continuous variables requires correlation coefficient of the paired data (correlation coefficient of pre-RFCA and post-RFCA scores in our case). The secondary analysis investigated whether the treatment success had any effect on the ΔPCS and ΔMCS. The ΔPCS and ΔMCS were extracted separately for the treatment success group and AF recurrence group from the individual studies. A meta-analysis of the independent group continuous data was performed since the treatment success and failure groups were independent of each other. The required combination of the raw data were as follows: (i) the ΔPCS and ΔMCS of each group, SD of the ΔPCS and ΔMCS of each group, and sample size of each group; and (ii) the ΔPCS and ΔMCS of each group, sample size of each group, and an independent *p*-value. For all analyses, the upper limit of the *p*-values were used whenever reported as a range (for example, a value of 0.001 was used for the calculation if the *p*-value was reported as <0.001).

A random effects model was used to calculate the overall effect size (weighted mean difference [WMD]), 95% confidence intervals, and *p*-values. The statistical heterogeneity was quantified with the *I*^*2*^ statistics using a fixed effect model. The possibility of a publication bias was evaluated through a funnel plot analysis. A Begg and Mazumdar’s rank correlation test and Egger’s regression intercept test were performed in addition to a visual estimation of the asymmetry. If a visual asymmetry of the funnel plot was suspected or the Begg’s and Egger’s test indicated the presence of a publication bias, Duval and Tweedie’s trim and fill method [17] was used to estimate the number of possible missing studies and calculate the adjusted overall effected size as if these missing studies were present. A fixed effect model was used to estimate the number of missing studies and a random effects model was used to calculate the adjusted overall effect size since previous reports indicated that fixed-random model performs better than fixed-fixed model or random-random model [18]. The influence of the individual studies was evaluated through a separate meta-analysis removing each individual study one at a time. A cumulative meta-analysis of the included studies ordered by publication year was also performed. The cumulative meta-analysis is a repetitive performance of the meta-analysis whenever a new study is added. Therefore, the cumulative meta-analysis sorted by publication year (the most remote study included first and most recent study last) might reveal temporal trends in the outcome measures. The present meta-analysis was performed under compliance with the PRISMA guidelines (S1 Fig). Statistical analyses were performed with the Comprehensive Meta-Analysis software (Biostat, Englewood, NJ, USA). A *p*-value less than 0.05 was considered to indicate statistical significance. The review protocol has not been registered.

## Results

### Search results

**Fig 1:**
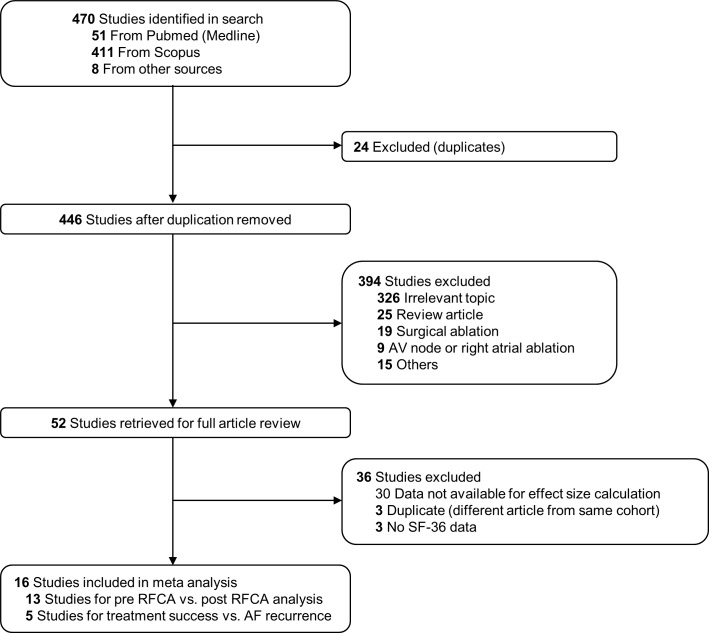
Study selection process. The flow diagram is depicted in accordance with the Preferred Reporting Items for Systematic reviews and Meta-Analyses guidelines. AF: atrial fibrillation; SF-36: short form-36; RFCA: radiofrequency catheter ablation.

A total of 470 studies were identified by the initial search strategy. The titles and abstracts were screened and 52 articles were retrieved for the full text review. After the full text review process and eligibility assessment, 16 articles meeting the inclusion criteria were selected for the final analysis (Fig 1) [19–34]. Among those 16 studies, 13 were available for the pre-RFCA vs. post-RFCA analysis and 5 studies for the treatment success group vs. AF recurrence group analysis. The baseline characteristics of the included studies are summarized in Tables 1 and 2. Thirteen studies used for the pre-RFCA vs. post-RFCA analysis consisted of 1,681 AF patients. All patients were resistant to at least 1 AAD. Only paroxysmal AF patients were evaluated in 7 studies. PVI was performed in all studies and an additional linear or complex fractionated atrial electrogram (CFAE) ablation was performed in 4 studies. The rate of a successful ablation (no AF recurrence) ranged between 56% to 89%. Five studies used for the treatment success vs. AF recurrence analysis consisted of 2,512 AF patients resistant to at least 1 AAD. There were two studies that evaluated only paroxysmal AF or long-standing persistent (≥ 1 year duration) AF. PVI was performed in all 5 studies. The treatment success rates were between 57% to 87%. The results of the Newcastle-Ottawa Scale are presented in S2 Table. Complication rate after RFCA varied between 0% to 26% and specific complications of each study are summarized in S3 Table. All studies included in the analysis used warfarin as an anticoagulant except for one study which used warfarin or dabigatran based on the operator’s discretion. Anticoagulation regimen of each study before and after RFCA is summarized in S4 Table.

**Table 1:** Baseline characteristics of the included studies: pre-RFCA vs. post-RFCA.

Source (Year)	Study design	Type of AF	Prior treatment with AAD	Mean duration of AF (year)	Procedure type	Successful ablation of AF	Follow up measurement of SF-36 (month)	Number of patients	Age (Year)	Mean LA diameter (mm)	Major exclusion criteria
Tada et al. (2003)	Observational study	Paroxysmal	Y	6.5 ± 6.8	SPVI	56%	6	50	58 ± 7	40 ± 7	NR
Pürerfellner et al. (2004)	Observational study	Paroxysmal	Y	6.3 ± 5.4	SPVI	89.3%	6	61	52.6 ± 10.8	42 ± 6	Significant underlying heart disease
Cha et al. (2008)	Observational study	Paroxysmal (52%), Persistent or Permanent (42%)	Y	6.4 ± 5.9	CPVI (57%), WACA (42%)	83.6%	12	432	54 ± 10	NR	NR
Carnlöf et al. (2010)	Observational study	Paroxysmal, Persistent	Y	NR	PVI	NR	6	34	53 ± 9	NR	NR
Wokhlu et al. (2010)	Observational study	Paroxysmal (51%), Persistent (35%), Long-standing (13%)	Y	6.6 ± 5.9	CPVI (22%), WACA (78%)	87%	24	323	55.9 ± 10.3	NR	NR
Reynolds et al. (2010)	Observational study*	Paroxysmal	Y	3 (2–8)	CPVI + Linear ablation, CFAE ablation, Cavotricuspid isthmus ablation (based on operator’s decision)	66%	3	97	55.5 ± 9.4	40.0 ± 1.1	Ejection fraction of less than 40%, previous ablation for AF, myocardial infarction within the previous 2 months, severe pulmonary disease
Pappone et al. (2011)	Observational study*	Paroxysmal	Y	6 ± 4	CPVA + Cavotricuspid isthmus ablation	72.7%	48	99	55 ± 10	40 ± 6	Persistent AF, Left atrial diameter > 65 mm, LVEF < 35%, heart failure symptoms
Höglund et al. (2013)	Observational study	Paroxysmal (50%), Persistent (48%), Long-standing (2%)	Y	NR	SPVI (34%), wide antral circumferential isolation (41%), isolation with the multi-polar catheter (25%)	62%	10	105	58 ± 9	44 ± 7	NR
Mantovan et al. (2013)	Observational study*	Paroxysmal (64%), Persistent (36%)	Y	7 ± 7	WACA (32%), WACA + CFAE (34%), CFAE (34%)	63.0%	12	100	57 ± 10	42 ± 6	Patients with permanent atrial fibrillation, patients who have previously undergone atrial fibrillation ablation, patients with left atrial size >55 mm
Sang et al. (2013)	Observational study	Parosyxmal	Y	7.5 ± 7.5	CPVI, Cavotricuspid isthmus ablation (if atrial flutter was documented)	72%	12	82	55.9 ± 6.1	39.0 ± 5.9	Previous nonpharmacological interventions for AF, New York Heart Association functional class III or IV, myocardial infarction, cardiac surgery or transient ischemic attack/stroke within the previous 6 months
Efremidis et al. (2014)	Observational study	Parosyxmal	Y	4.9 ± 4.7	WACA	71.9%	6	57	56.9 ± 12.2	40.4 ± 4.7	Left atrial diameter >50 mm, systolic heart failure, persistent AF
Natale et al. (2014)	Observational study*	Parosyxmal	Y	4.0 (1.4–7.1)	CPVI + Linear ablation, CFAE ablation, Cavotricuspid isthmus ablation (based on operator’s decision)	74.0%	12	117	58.3 ± 10.9	38.5 ± 5.6	AF of more than 30 days in duration, ejection fraction <40%, previous AF ablation, New York Heart Association functional class III or class IV, severe pulmonary disease
Wynn et al. (2015)	Observational study*	Paroxysmal (39%), Persistent (61%)	Y	5.5 ± 4.0	WACA (49%), WACA + linear ablation (51%)	64.8%	12	122	61.9 ± 10.5	43 ± 6	Long-standing (>12 months) persistent AF, previous AF ablation, documented typical atrial flutter

*: These studies were originally randomized clinical trials. However, since we used pre and post data of only RFCA arm, we classified these studies as observational studies.

AAD: antiarrhythmic drugs; AF: atrial fibrillation; CFAE: complex fractionated atrial electrogram; CPVA: circumferential pulmonary vein ablation; CPVI: circumferential pulmonary vein isolation; LA: left atrium; LVEF: left ventricular ejection fraction; NR: not reported; PVI: pulmonary vein isolation; RFCA: radiofrequency catheter ablation; SF-36: short form-36; SPVI: segmental pulmonary vein isolation; WACA: wide area catheter ablation.

**Table 2:** Baseline characteristics of the included studies: Treatment success group vs. AF recurrence group.

Source (Year)	Study design	Type of AF	Prior treatment with AAD	Mean duration of AF (year)	Procedure type	Successful ablation of AF	Follow up measurement of SF-36 (month)	Number of patients	Age (Year)	Mean LA diameter (mm)	Major exclusion criteria
Wokhlu et al. (2010)	Observational study	Paroxysmal (51%), Persistent (35%), Long-standing (13%)	Y	6.6 ± 5.9	CPVI(22%), WACA(78%)	87%	24	323	55.9 ± 10.3	NR	NR
Mohanty et al. (2012)	Observational study	Paroxysmal (29.3%), Persistent (26.3%), Long-standing persistent (44.4%)	Y	NR	PVI + Linear ablation, CFAE ablation, superior vena cava ablation	66.0%	12	1496	62.6 ± 9.4	43.2 ± 7.5	NR
Sang et al. (2013)	Observational study	Parosyxmal	Y	7.5 ± 7.5	CPVI, Cavotricuspid isthmus ablation (if atrial flutter was documented)	72%	12	82	55.9 ± 6.1	39.0 ± 5.9	Previous nonpharmacological interventions for AF, New York Heart Association functional class III or IV, myocardial infarction, cardiac surgery or transient ischemic attack/stroke within the previous 6 months
Gu et al. (2013)	Observational study	Paroxysmal (34.7%), Persistent (34.4%), Long-standing persistent (30.9%)	Y	4.9 ± 1.1	CPVI + Linear ablation, CFAE ablation, Cavotricuspid isthmus ablation (based on operator’s decision)	69.8%	24	550	64.2 ± 8.7	46.2 ± 3.3	NR
Mohanty et al. (2014)	Observational study	Long-standing persistent	Y	6.4 (2.5–9)	PVI + CFAE ablation + non-pulmonary vein trigger ablation	57%	12	61	62 ± 13	43.8 ± 7.8	NR

AAD: antiarrhythmic drugs; AF: atrial fibrillation; CFAE: complex fractionated atrial electrogram; CPVI: circumferential pulmonary vein isolation; LA: left atrium; LVEF: left ventricular ejection fraction; NR: not reported; PVI: pulmonary vein isolation; RFCA: radiofrequency catheter ablation; SF-36: short form-36; WACA: wide area catheter ablation.

### Pre-RFCA vs. post-RFCA

**Fig 2:**
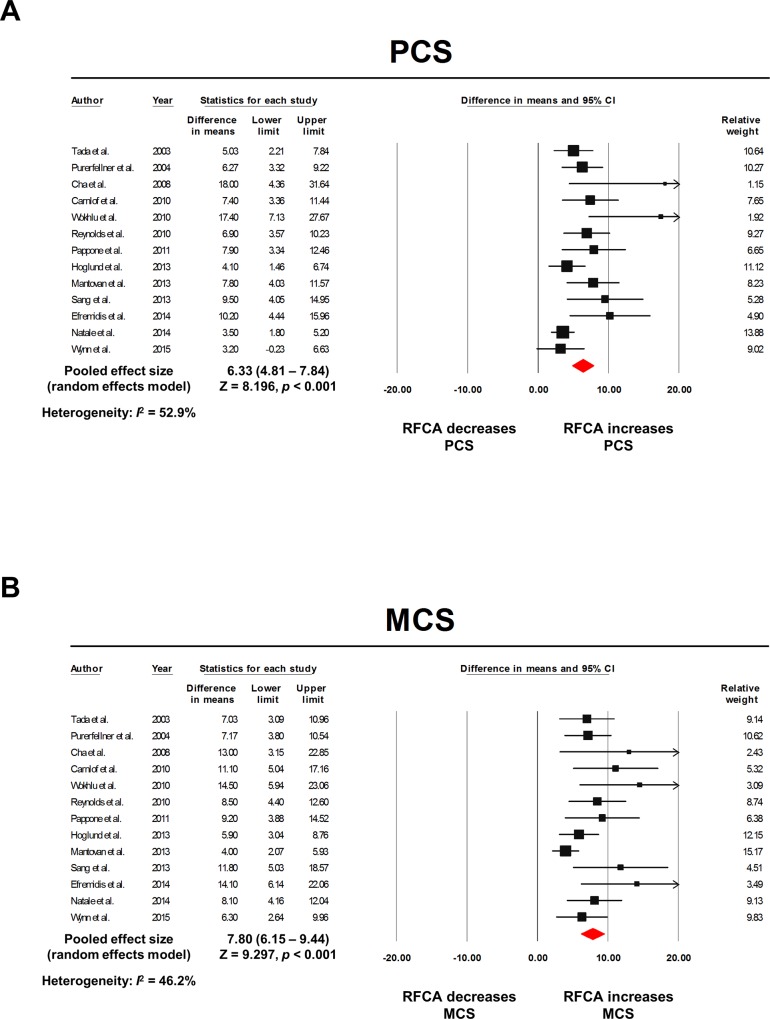
Forest plots: pre-RFCA vs. post-RFCA. The pooled WMD of PCS **(A)** and MCS **(B)** are presented. The size of the black squares corresponds to the weight of each study included. The overall effect size was calculated with a random effects model. The raw data extracted from each study are described in Table 3. CI: confidence intervals; MCS: mental component summary score; PCS: physical component summary score; RFCA: radiofrequency catheter ablation; WMD: weighted mean difference.

**Fig 3:**
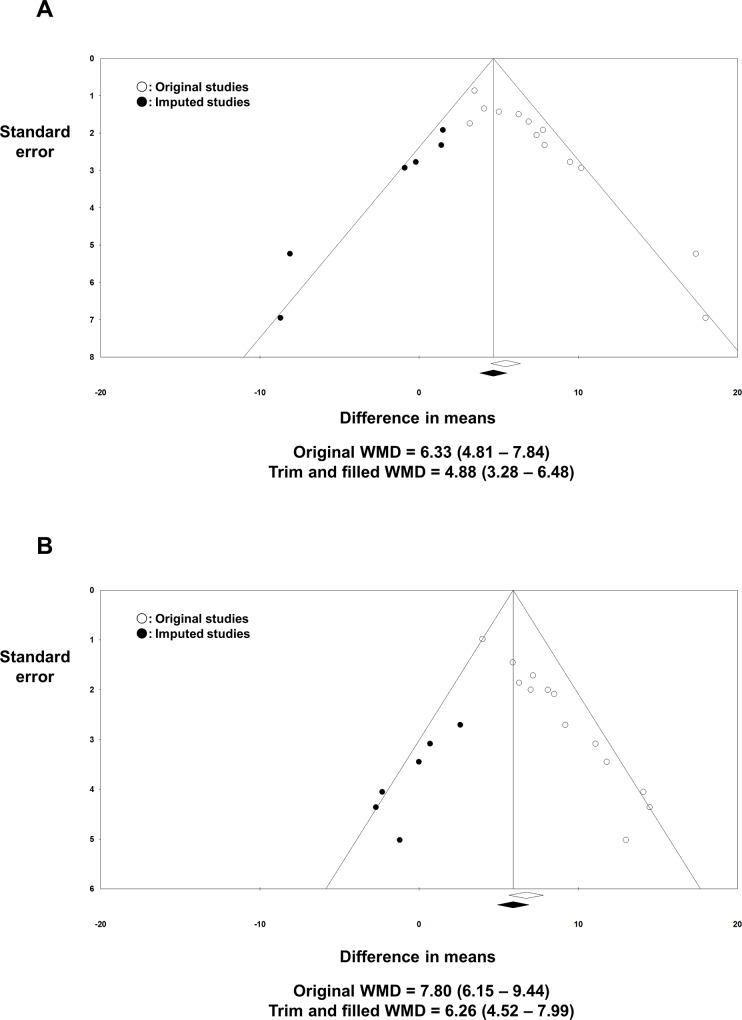
Funnel plots: pre-RFCA vs. post-RFCA. Possible missing studies are imputed in addition to the original studies to adjust for the possible publication bias. The results of the trim and filled WMD of PCS **(A)** and MCS **(B)** are presented. MCS: mental component summary score; PCS: physical component summary score; WMD: weighted mean difference.

The raw data extracted from the individual studies for the effect size calculation are summarized in Table 3. The pooled model showed significant improvements in both the PCS and MCS after the RFCA. The WMD was 6.33 (4.81–7.84; *I*^*2*^ = 52.9%; *p* < 0.001; Fig 2A) for the PCS and 7.80 (6.15–9.44; *I*^*2*^ = 46.2%; *p* < 0.001; Fig 2B) for the MCS. Visual asymmetries were suspected in the funnel plot analysis in both the PCS and MCS, which was supported by the Begg’s and Egger’s test (*p* < 0.001; S2 Fig). To adjust for the possible confounding effects of a publication bias, a trim and fill method was utilized. There were no significant visual asymmetries in the trim and filled funnel plots and the adjusted WMD was 4.88 (3.28–6.48; Fig 3A) for the PCS and 6.26 (4.52–7.99; Fig 3B) for the MCS. In the meta-analysis with one study removed, there was no individual study that substantially influenced the overall effect size for both the PCS and MCS (S3 Fig). The cumulative meta-analysis did not show any temporal trends for the degree of improvement in both the PCS and MCS after RFCA (S4 Fig).

**Table 3:** Raw data extracted from the individual studies: pre-RFCA vs. post-RFCA.

Source (Year)	PCS							MCS						
	Mean PCS (Pre)	Mean PCS (Post)	n	Paired *p*-value	ΔPCS	n	Paired *p*-value	Mean MCS (Pre)	Mean MCS (Post)	n	Paired *p*-value	ΔMCS	n	Paired *p*-value
Tada et al. (2003)	43.8	48.8	50	< 0.001				40.8	47.8	50	< 0.001			
Pürerfellner et al. (2004)	45.4	51.7	61	< 0.0001				44.5	51.7	61	< 0.0001			
Cha et al. (2008)	59.0	77.0	432	< 0.01				66.0	79.0	432	< 0.01			
Carnlöf et al. (2010)	40.1	47.5	36	< 0.001				38.2	49.3	36	< 0.001			
Wokhlu et al. (2010)	58.8	76.2	323	< 0.001				65.3	79.8	323	< 0.001			
Reynolds et al. (2010)					6.9	97	< 0.0001					8.5	97	< 0.0001
Pappone et al. (2011)	44.4	52.3	99	< 0.001				43.7	52.9	99	< 0.001			
Höglund et al. (2013)	39.8	43.9	105	0.003				41.7	47.6	105	< 0.0001			
Mantovan et al. (2013)	47.9	55.7	100	< 0.0001				33.4	37.4	100	0.0001			
Sang et al. (2013)					9.5	82	< 0.001					11.8	82	< 0.001
Efremidis et al. (2014)	68.0	78.2	57	< 0.001				65.1	79.2	57	< 0.001			
Natale et al. (2014)					3.5	117	< 0.0001					8.1	117	< 0.0001
Wynn et al. (2015)	44.7	47.9	122	0.07				45.7	52.0	122	< 0.001			

MCS: mental component summary score; PCS: physical component summary score; RFCA: radiofrequency catheter ablation.

**Fig 4:**
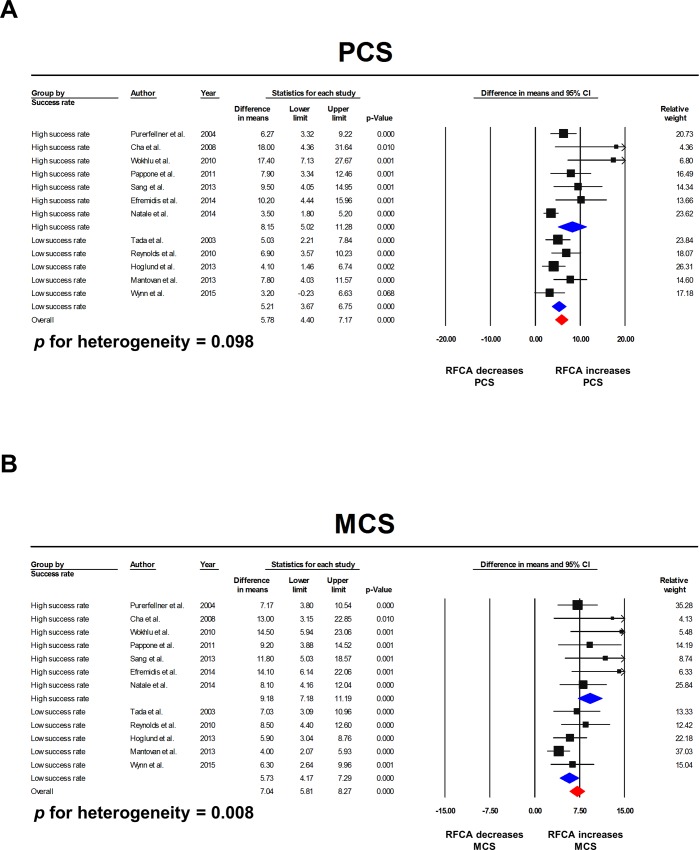
Subgroup analysis according to the treatment success rate. Studies that reported a treatment success rate over 70% showed a trend toward a better improvement in the PCS **(A)** and a significantly better improvement in the MCS **(B)**. CI: confidence intervals; MCS: mental component summary score; PCS: physical component summary score; RFCA: radiofrequency catheter ablation.

Since the studies included in the meta-analysis showed significant variations in the treatment success rate, we performed a subgroup analysis between the high success rate group (≥ 70%) vs. low success rate group (< 70%). In the PCS, the pooled WMD was 8.15 (5.02–11.28) for the high success rate group and 5.21 (3.67–6.75) for the low success rate group. The *p*-value for between group heterogeneity was 0.098 (Fig 4A). The MCS had a more obvious difference between the 2 groups. The pooled WMD was 9.18 (7.18–11.19) for the high success rate group and 5.73 (4.17–7.29) for the low success rate group (*p =* 0.008; Fig 4B). No statistically significant subgroup differences were identified in terms of the AF type (*p* = 0.705 for PCS, 0.474 for MCS; Figure A in S5 Fig file) and procedure type (*p* = 0.159 for PCS, 0.128 for MCS; Figure B in S5 Fig file).

### Treatment success vs. AF recurrence

**Fig 5:**
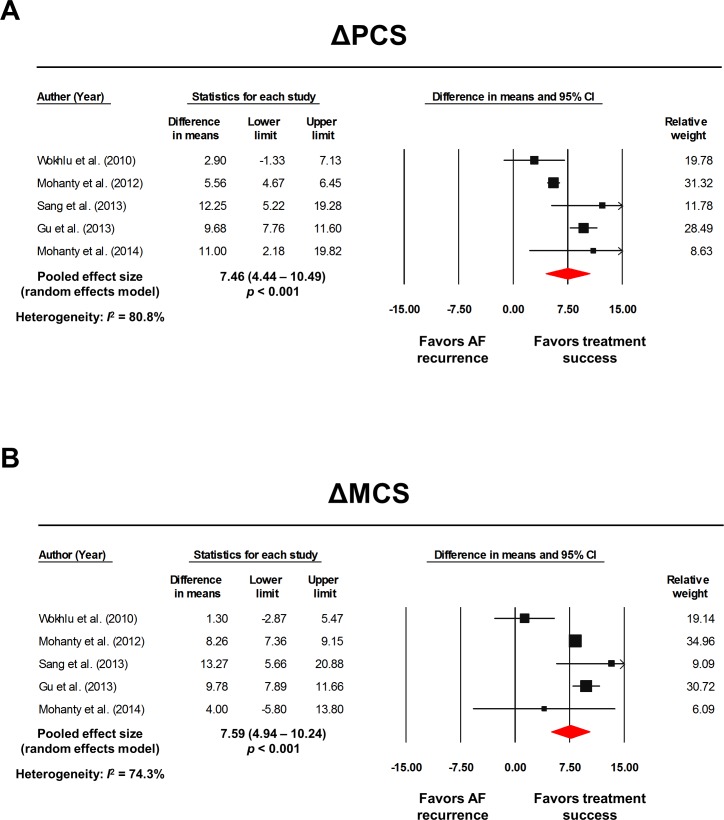
Forest plots: treatment success vs. AF recurrence. The pooled WMD of ΔPCS **(A)** and ΔMCS **(B)** are presented. The size of the black squares corresponds to the weight of each study included. The overall effect size was calculated with a random effects model. The raw data extracted from each study are described in Table 4. AF: atrial fibrillation; CI: confidence intervals; MCS: mental component summary score; PCS: physical component summary score; WMD: weighted mean difference.

**Fig 6:**
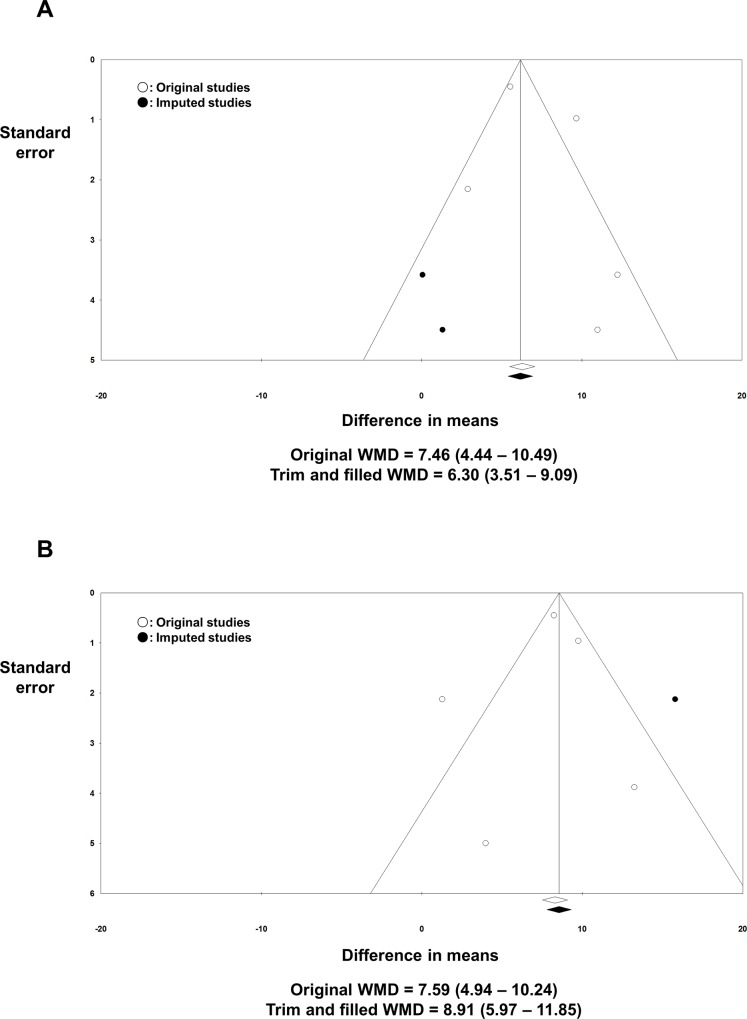
Funnel plots: treatment success vs. AF recurrence. A visual asymmetry was suspected in the funnel plot analysis of the ΔPCS **(A)** and ΔMCS **(B)**. After applying the trim and fill method, possible missing studies were imputed. Original and adjusted WMDs are presented. AF: atrial fibrillation; MCS: mental component summary score; PCS: physical component summary score; WMD: weighted mean difference.

Table 4 summarizes the raw data extracted from the individual studies to calculate the overall effect size. The baseline PCS and MCS would directly affect the post-RFCA PCS and MCS. Furthermore, there is a possibility that the baseline PCS and MCS might also influence the treatment success rate. Consequently, comparing the post-RFCA PCS and MCS between the non-recur group and recur group might result in an erroneous interpretation. In order to resolve this problem, we compared the ΔPCS and ΔMCS between the non-recur group and recur group. The pooled analysis revealed a significant difference in the WMD for both the ΔPCS and ΔMCS between the non-recur group and recur group. The WMD was 7.46 (4.44–10.49; *I*^*2*^ = 80.8%; *p* < 0.001; Fig 5A) for the ΔPCS and 7.59 (4.94–10.24; *I*^*2*^ = 74.3%; *p* < 0.001; Fig 5B) for the ΔMCS, all favoring the non-recur group. A visual analysis of the funnel plot of the ΔPCS and ΔMCS indicated a possible publication bias although both the Begg’s (*p =* 0.624, 0.624 respectively) and Egger’s (*p* = 0.450, 0.694 respectively) tests were not statistically significant. The trim and fill method was applied and the adjusted WMD was 6.30 (3.51–9.09; Fig 6A) for ΔPCS and 8.91 (5.97–11.85; Fig 6B) for ΔMCS. There was no significant change in the pooled WMD of the ΔPCS & ΔMCS after removing each study one at a time (S6 Fig).

**Table 4:** Raw data extracted from the individual studies: Treatment success group vs. AF recurrence group.

Source (Year)	PCS							MCS						
	Sinus rhythm			AF recurrence			Independent *p*-value	Sinus rhythm			AF recurrence			Independent *p*-value
	ΔPCS	SD	n	ΔPCS	SD	n		ΔMCS	SD	n	ΔMCS	SD	n	
Wokhlu et al. (2010)	16.6	17.4	224	13.7	18.9	99		13.1	16.6	224	11.8	19.8	99	
Mohanty et al. (2012)	6.8	3.8	988	1.2	13.2	508		11.0	6.6	988	2.7	11.0	508	
Sang et al. (2013)	11.4		53	-0.9		29	< 0.001	13.2		53	-0.1		29	< 0.001
Gu et al. (2013)	11.0	11.3	384	1.3	8.5	166		9.9	10.7	384	0.1	9.6	166	
Mohanty et al. (2014)	8.0	16.0	36	-3.0	19.0	25		6.0	20.0	36	2.0	18.0	25	

AF: atrial fibrillation; MCS: mental component summary score; PCS: physical component summary score; SD: standard deviation.

## Discussion

We performed a systematic review and meta-analysis that evaluated the relationship between RFCA and the patient QoL in AF patients. To the best of our knowledge, this is the first meta-analysis with a focus on investigating whether performing RFCA and achieving treatment success are associated with an improved QoL in AF patients. The principal findings of our study are as follows: (i) RFCA of the pulmonary veins significantly improved both the PCS and MCS. The WMD (pre-RFCA vs. post-RFCA) of the PCS and MCS were 6.33 and 7.80, respectively; (ii) Patients who were successfully treated with RFCA had a greater improvement in both the PCS and MCS compared to those who had AF recurrences. The WMD between the treatment success group and AF recurrence group was 7.46 and 7.59 for the ΔPCS and ΔMCS, respectively.

A 5 point increase in the SF-36 score is known to represent a clinically significant improvement [21, 35]. Of note, the PCS and MCS are T-scores with a mean of 50 and SD of 10. Since the WMD ranged between 6.33 and 7.80, our analysis suggests that RFCA significantly improves the PCS and MCS in the AF patients and the treatment success is important for the QoL outcomes. In this meta-analysis, the overall effect size (WMD) was actively adjusted through a trim and fill strategy whenever a publication bias was suspected. The influences of the individual studies were also searched thoroughly.

### RFCA and the patient QoL

The prevalence rate of AF is estimated to be 1–2% of the general population [36]. In 2001, Go et al. anticipated that the number of AF patients in the United States would be 5.6 million by the year 2050 [37]. In 2006, however, Miyasaka et al. reported that the age adjusted incidence rate of AF had significantly increased during 1980 to 2000 in Olmsted County and based on this epidemiologic shift, the total number of AF patients in the United Stated was estimated to be 10 million in 2050 [38]. This widely prevalent disease, which is anticipated to be even more prevalent in the near future, is associated with an increased long-term risk of stroke, HF, and all-cause mortality [36, 39, 40]. Importantly, AF is not only associated with the long-term adverse clinical outcomes, but also with a significant impairment in the QoL [41, 42]. Dorian et al. reported that all 8 domains of the SF-36 were lower in the AF patients than healthy controls by 1.3 to 2.0 standard deviation units [41]. Furthermore, most patients with AF come to the clinics for the first time because of their symptomatic discomfort such as palpitations, dyspnea, and general fatigue, and their major interest is the elimination of AF-related symptoms. Unfortunately, antiarrhythmic drug treatment has not shown a consistent benefit in terms of improving the patient QoL [42, 43].

RFCA is rapidly emerging as an alternative treatment option for AF patients and has shown a reduced recurrence rate compared to AAD [4, 5, 28, 44]. Whether RFCA improves the QoL in AF patients has also been extensively investigated. Among the several instruments to measure the QoL in AF patients, the SF-36 is the most widely used and validated instrument [12, 14]. Although several studies assessed the impact of RFCA on QoL in AF patients using the SF-36, substantial proportions of studies have mainly focused on the treatment success rate rather than the improvements in the SF-36. Furthermore, the degree of change in the SF-36 score has varied from study to study and there have been no systematic reviews or meta-analyses of the SF-36 in AF patients undergoing RFCA. Since the primary purpose of performing RFCA in AF patients is to improve their QoL and to reduce AF-related symptoms, it is crucial to demonstrate whether RFCA improves the QoL, and if it does, elucidate the degree of improvement. Our data might provide a valid information to clinicians working in the field of AF.

### Placebo effect vs. true treatment effect

Our meta-analysis and the individual studies included in the analysis suggested that RFCA improves the PCS and MCS of AF patients. However, the analysis is a pre and post RFCA comparison and a procedure related placebo effect cannot be completely ruled out because data of a sham procedure is not available. In order to exclude the possibility of procedure related placebo effect, comparing the treatment effect according to procedural success might be useful. We analyzed the WMD of the ΔPCS and ΔMCS in the treatment success group and AF recurrence group. Both the ΔPCS and ΔMCS were significantly higher in the treatment success group. Since the result of the procedure is associated with higher ΔPCS and ΔMCS, the effect of RFCA is more likely a true treatment effect rather than a placebo effect.

Pre-RFCA PCS and MCS might affect the treatment success rate. It is probable that patients with higher PCS and MCS are more likely to be in good general condition, have a shorter history of AF (and consequently smaller left atrial size), and lesser AF burden, all of which would lead to a higher treatment success rate. Consequently, the patients in the treatment success group are more likely to have higher post-RFCA PCS and MCS (since they are more likely to have better pre-RFCA scores) compared to the AF recurrence group which suggests that post-RFCA PCS and MCS do not fully reflect the treatment effect of RFCA. In order to resolve this problem, we compared the ΔPCS and ΔMCS rather than the post-RFCA PCS and MCS. By comparing the degree of improvement, we demonstrated that successful ablation is important for improving the QoL in AF patients.

### Different types of AF and RFCA procedures

In general, AF is categorized into paroxysmal (< 7days), persistent (≥ 7days), long-standing (≥ 1 year), and permanent [36]. Paroxysmal AF is more suitable for catheter ablation since most of abnormal electrical signals originate from the pulmonary veins [45]. AF other than paroxysmal, however, is more difficult to manage and the results of RFCA are less favorable [34, 46]. In order to overcome this problem, several ablation techniques, such as creation of linear lesions and focal ablation of complex activity, are often used in addition to PVI. A recent randomized trial, however, showed that adding linear ablation or focal ablation of complex activity to PVI offered no benefit in terms of AF recurrence [45]. Our subgroup analysis also indicated that linear ablation or focal ablation of complex activity performed in addition to PVI did not improve the PCS or MCS. However, all studies included in our analysis were observational studies and addition of linear ablation or focal ablation was based on the operator’s decision rather than obligational in 2 studies. Our results should be interpreted with caution.

### Limitations

There are several limitations to this analysis that should be considered. First, the studies included for the meta-analysis had different protocols such as the follow up duration and exact type of ablation procedure. Second, this was a study-level meta-analysis and we were not able to adjust for the patient-level confounders such as age, sex, body mass index, diabetes, left atrial size, etc. Consequently, a subgroup analysis of interest was limited. Third, the PCS and MCS were not available in a substantial amount of studies, and rather they only reported 8 components of the SF-36. In addition, several studies did not report a paired *p*-value of the PCS and MCS (pre-RFCA vs. post-RFCA). In studies that reported only the mean, SD, and sample size, it was not possible to calculate the effect size since a pre and post correlation, which is necessary for the effect size calculation, is almost never reported in the standard medical journals. Finally, we were not able to evaluate the clinical benefit of cryoablation in addition to RFCA, since there was no available article to do so.

## Conclusion

RFCA is likely to be associated with improvements in the PCS and MCS in drug-refractory AF patients. Patients without AF recurrence after RFCA had a greater improvement in both the PCS and MCS compared to those who experienced AF recurrence.

## Supporting Information

S1 Fig Checklist of the PRISMA guidelines. PRISMA: Preferred Reporting Items for Systematic reviews and Meta-Analyses.
(DOCX)

S2 Fig Funnel plot analysis: pre-RFCA vs. post-RFCA. Publication bias was suspected in both the PCS **(A)** and MCS **(B)**. MCS: mental component summary score; PCS: physical component summary score; RFCA: radiofrequency catheter ablation; WMD: weighted mean difference.
(DOCX)

S3 Fig Influence of the individual studies: pre-RFCA vs. post-RFCA. No significant change in the overall WMD was noted in both the PCS **(A)** and MCS **(B)** whenever each study was removed. CI: confidence intervals; MCS: mental component summary score; PCS: physical component summary score; RFCA: radiofrequency catheter ablation; WMD: weighted mean difference.
(DOCX)

S4 Fig Cumulative meta-analysis: pre-RFCA vs. post-RFCA. The cumulative meta-analysis showed no definite temporal trends in the efficacy of RFCA in both the PCS **(A)** and MCS **(B)**. CI: confidence intervals; PCS: physical component summary score; MCS: mental component summary score; RFCA: radiofrequency catheter ablation.
(DOCX)

S5 Fig Subgroup analysis according to the composition of AF type and procedure type. **(A)** There was no significant difference in the pooled WMD of the PCS and MCS between studies including only paroxysmal AF vs. studies including various types of AF. **(B)** Performing additional procedures such as linear ablation or CFAE ablation was not associated with improved outcomes compared to performing pulmonary vein isolation only. AF: atrial fibrillation; CFAE: complex fractionated atrial electrogram; MCS: mental component summary score; PCS: physical component summary score; RFCA: radiofrequency catheter ablation; WMD: weighted mean difference.
(DOCX)

S6 Fig Influence of the individual studies: treatment success vs. AF recurrence. No significant change in the overall WMD was noted in both the ΔPCS **(A)** and ΔMCS **(B)** whenever each study was removed. AF: atrial fibrillation; CI: confidence intervals; MCS: mental component summary score; PCS: physical component summary score; WMD: weighted mean difference.
(DOCX)

S1 Table Search strategy. (DOCX)

S2 Table The Newcastle-Ottawa scale for assessing the quality of 16 non-randomized studies included in the meta-analysis. (DOCX)

S3 Table Procedure related complications of each study. (DOCX)

S4 Table Anticoagulation therapy before and after RFCA. (DOCX)

## Abbreviations

AAD: antiarrhythmic drugs
AF: atrial fibrillation
CFAE: complex fractionated atrial electrogram
MCS: mental component summary score
PCS: physical component summary score
PRISMA: Preferred Reporting Items for Systematic reviews and Meta-Analyses
PVI: pulmonary vein isolation
QoL: quality of life
RFCA: radiofrequency catheter ablation
SD: standard deviation
SF-36: short form-36
WMD: weighted mean difference.

## References

[1] Wyse DG, Waldo AL, DiMarco JP, Domanski MJ, Rosenberg Y, Schron EB, et al A comparison of rate control and rhythm control in patients with atrial fibrillation. N Engl J Med. 2002;347(23):1825–1833. 12466506 10.1056/NEJMoa021328

[2] Corley SD, Epstein AE, DiMarco JP, Domanski MJ, Geller N, Greene HL, et al Relationships between sinus rhythm, treatment, and survival in the Atrial Fibrillation Follow-Up Investigation of Rhythm Management (AFFIRM) Study. Circulation. 2004;109(12):1509–1513. 15007003 10.1161/01.CIR.0000121736.16643.11

[3] Pappone C, Augello G, Sala S, Gugliotta F, Vicedomini G, Gulletta S, et al A randomized trial of circumferential pulmonary vein ablation versus antiarrhythmic drug therapy in paroxysmal atrial fibrillation: the APAF Study. J Am Coll Cardiol. 2006;48(11):2340–2347. 17161267 10.1016/j.jacc.2006.08.037

[4] Jais P, Cauchemez B, Macle L, Daoud E, Khairy P, Subbiah R, et al Catheter ablation versus antiarrhythmic drugs for atrial fibrillation: the A4 study. Circulation. 2008;118(24):2498–2505. 10.1161/CIRCULATIONAHA.108.772582 19029470

[5] Wilber DJ, Pappone C, Neuzil P, De Paola A, Marchlinski F, Natale A, et al Comparison of antiarrhythmic drug therapy and radiofrequency catheter ablation in patients with paroxysmal atrial fibrillation: a randomized controlled trial. JAMA. 2010;303(4):333–340. 10.1001/jama.2009.2029 20103757

[6] Packer DL, Kowal RC, Wheelan KR, Irwin JM, Champagne J, Guerra PG, et al Cryoballoon ablation of pulmonary veins for paroxysmal atrial fibrillation: first results of the North American Arctic Front (STOP AF) pivotal trial. J Am Coll Cardiol. 2013;61(16):1713–1723. 10.1016/j.jacc.2012.11.064 23500312

[7] Oral H, Pappone C, Chugh A, Good E, Bogun F, Pelosi F Jr., et al Circumferential pulmonary-vein ablation for chronic atrial fibrillation. N Engl J Med. 2006;354(9):934–941. 16510747 10.1056/NEJMoa050955

[8] Morillo CA, Verma A, Connolly SJ, Kuck KH, Nair GM, Champagne J, et al Radiofrequency ablation vs antiarrhythmic drugs as first-line treatment of paroxysmal atrial fibrillation (RAAFT-2): a randomized trial. JAMA. 2014;311(7):692–700. 10.1001/jama.2014.467 24549549

[9] Cosedis Nielsen J, Johannessen A, Raatikainen P, Hindricks G, Walfridsson H, Kongstad O, et al Radiofrequency ablation as initial therapy in paroxysmal atrial fibrillation. N Engl J Med. 2012;367(17):1587–1595. 10.1056/NEJMoa1113566 23094720

[10] Wazni OM, Marrouche NF, Martin DO, Verma A, Bhargava M, Saliba W, et al Radiofrequency ablation vs antiarrhythmic drugs as first-line treatment of symptomatic atrial fibrillation: a randomized trial. JAMA. 2005;293(21):2634–2640. 15928285 10.1001/jama.293.21.2634

[11] Shi LZ, Heng R, Liu SM, Leng FY. Effect of catheter ablation versus antiarrhythmic drugs on atrial fibrillation: A meta-analysis of randomized controlled trials. Exp Ther Med. 2015;10(2):816–822. 26622399 10.3892/etm.2015.2545 PMC4508979

[12] Reynolds MR, Ellis E, Zimetbaum P. Quality of life in atrial fibrillation: measurement tools and impact of interventions. J Cardiovasc Electrophysiol. 2008;19(7):762–768. 10.1111/j.1540-8167.2007.01091.x 18266667 PMC2574796

[13] Koci F, Forbes P, Mansour MC, Heist EK, Singh JP, Ellinor PT, et al New classification scheme for atrial fibrillation symptom severity and burden. Am J Cardiol. 2014;114(2):260–265. 10.1016/j.amjcard.2014.04.032 24878121

[14] Aliot E, Botto GL, Crijns HJ, Kirchhof P. Quality of life in patients with atrial fibrillation: how to assess it and how to improve it. Europace. 2014;16(6):787–796. 10.1093/europace/eut369 24469433

[15] Ware JE Jr., Sherbourne CD. The MOS 36-item short-form health survey (SF-36). I. Conceptual framework and item selection. Med Care. 1992;30(6):473–483. 1593914

[16] Liberati A, Altman DG, Tetzlaff J, Mulrow C, Gotzsche PC, Ioannidis JP, et al The PRISMA statement for reporting systematic reviews and meta-analyses of studies that evaluate health care interventions: explanation and elaboration. Ann Intern Med. 2009;151(4):W65–94. 19622512 10.7326/0003-4819-151-4-200908180-00136

[17] Duval S, Tweedie R. Trim and fill: A simple funnel-plot-based method of testing and adjusting for publication bias in meta-analysis. Biometrics. 2000;56(2):455–463. 10877304 10.1111/j.0006-341x.2000.00455.x

[18] Peters JL, Sutton AJ, Jones DR, Abrams KR, Rushton L. Performance of the trim and fill method in the presence of publication bias and between-study heterogeneity. Stat Med. 2007;26(25):4544–4562. 17476644 10.1002/sim.2889

[19] Tada H, Naito S, Kurosaki K, Ueda M, Ito S, Shinbo G, et al Segmental pulmonary vein isolation for paroxysmal atrial fibrillation improves quality of life and clinical outcomes. Circ J. 2003;67(10):861–865. 14578620 10.1253/circj.67.861

[20] Purerfellner H, Martinek M, Aichinger J, Nesser HJ, Kempen K, Janssen JP. Quality of life restored to normal in patients with atrial fibrillation after pulmonary vein ostial isolation. Am Heart J. 2004;148(2):318–325. 15309003 10.1016/j.ahj.2004.03.036

[21] Cha YM, Friedman PA, Asirvatham SJ, Shen WK, Munger TM, Rea RF, et al Catheter ablation for atrial fibrillation in patients with obesity. Circulation. 2008;117(20):2583–2590. 10.1161/CIRCULATIONAHA.107.716712 18474813

[22] Carnlof C, Insulander P, Pettersson PH, Jensen-Urstad M, Fossum B. Health-related quality of life in patients with atrial fibrillation undergoing pulmonary vein isolation, before and after treatment. Eur J Cardiovasc Nurs. 2010;9(1):45–49. 10.1016/j.ejcnurse.2009.09.002 19825514

[23] Wokhlu A, Monahan KH, Hodge DO, Asirvatham SJ, Friedman PA, Munger TM, et al Long-term quality of life after ablation of atrial fibrillation the impact of recurrence, symptom relief, and placebo effect. J Am Coll Cardiol. 2010;55(21):2308–2316. 10.1016/j.jacc.2010.01.040 20488300

[24] Reynolds MR, Walczak J, White SA, Cohen DJ, Wilber DJ. Improvements in symptoms and quality of life in patients with paroxysmal atrial fibrillation treated with radiofrequency catheter ablation versus antiarrhythmic drugs. Circ Cardiovasc Qual Outcomes. 2010;3(6):615–623. 10.1161/CIRCOUTCOMES.110.957563 20940250

[25] Pappone C, Vicedomini G, Augello G, Manguso F, Saviano M, Baldi M, et al Radiofrequency catheter ablation and antiarrhythmic drug therapy: a prospective, randomized, 4-year follow-up trial: the APAF study. Circ Arrhythm Electrophysiol. 2011;4(6):808–814. 10.1161/CIRCEP.111.966408 21946315

[26] Hoglund N, Ronn F, Tollefsen T, Jensen SM, Kesek M. U22 protocol as measure of symptomatic improvement after catheter ablation of atrial fibrillation. Ups J Med Sci. 2013;118(4):240–246. 10.3109/03009734.2013.821190 24102147 PMC4190885

[27] Mantovan R, Macle L, De Martino G, Chen J, Morillo CA, Novak P, et al Relationship of quality of life with procedural success of atrial fibrillation (AF) ablation and postablation AF burden: substudy of the STAR AF randomized trial. Can J Cardiol. 2013;29(10):1211–1217. 10.1016/j.cjca.2013.06.006 23988341

[28] Sang CH, Chen K, Pang XF, Dong JZ, Du X, Ma H, et al Depression, anxiety, and quality of life after catheter ablation in patients with paroxysmal atrial fibrillation. Clin Cardiol. 2013;36(1):40–45. 10.1002/clc.22039 22777577 PMC6649502

[29] Efremidis M, Letsas KP, Lioni L, Giannopoulos G, Korantzopoulos P, Vlachos K, et al Association of quality of life, anxiety, and depression with left atrial ablation outcomes. Pacing Clin Electrophysiol. 2014;37(6):703–711. 10.1111/pace.12420 24809737

[30] Natale A, Reddy VY, Monir G, Wilber DJ, Lindsay BD, McElderry HT, et al Paroxysmal AF catheter ablation with a contact force sensing catheter: results of the prospective, multicenter SMART-AF trial. J Am Coll Cardiol. 2014;64(7):647–656. 10.1016/j.jacc.2014.04.072 25125294

[31] Wynn G, Panikker S, Morgan M, Hall M, Waktare J, Markides V, et al Biatrial linear ablation in sustained nonpermanent AF: Results of the substrate modification with ablation and antiarrhythmic drugs in nonpermanent atrial fibrillation trial. Heart Rhythm. 2015. 10.1016/j.hrthm.2015.10.006 26455343

[32] Mohanty S, Mohanty P, Di Biase L, Bai R, Pump A, Santangeli P, et al Impact of metabolic syndrome on procedural outcomes in patients with atrial fibrillation undergoing catheter ablation. J Am Coll Cardiol. 2012;59(14):1295–1301. 10.1016/j.jacc.2011.11.051 22464257

[33] Gu J, Liu X, Tan H, Zhou L, Jiang W, Wang Y, et al Impact of chronic obstructive pulmonary disease on procedural outcomes and quality of life in patients with atrial fibrillation undergoing catheter ablation. J Cardiovasc Electrophysiol. 2013;24(2):148–154. 10.1111/j.1540-8167.2012.02448.x 23066893

[34] Mohanty S, Santangeli P, Mohanty P, Di Biase L, Holcomb S, Trivedi C, et al Catheter ablation of asymptomatic longstanding persistent atrial fibrillation: impact on quality of life, exercise performance, arrhythmia perception, and arrhythmia-free survival. J Cardiovasc Electrophysiol. 2014;25(10):1057–1064. 10.1111/jce.12467 24903064

[35] Stewart AL, Greenfield S, Hays RD, Wells K, Rogers WH, Berry SD, et al Functional status and well-being of patients with chronic conditions. Results from the Medical Outcomes Study. JAMA. 1989;262(7):907–913. 2754790

[36] Fuster V, Ryden LE, Cannom DS, Crijns HJ, Curtis AB, Ellenbogen KA, et al ACC/AHA/ESC 2006 Guidelines for the Management of Patients with Atrial Fibrillation: a report of the American College of Cardiology/American Heart Association Task Force on Practice Guidelines and the European Society of Cardiology Committee for Practice Guidelines (Writing Committee to Revise the 2001 Guidelines for the Management of Patients With Atrial Fibrillation): developed in collaboration with the European Heart Rhythm Association and the Heart Rhythm Society. Circulation. 2006;114(7):e257–354. 16908781 10.1161/CIRCULATIONAHA.106.177292

[37] Go AS, Hylek EM, Phillips KA, Chang Y, Henault LE, Selby JV, et al Prevalence of diagnosed atrial fibrillation in adults: national implications for rhythm management and stroke prevention: the AnTicoagulation and Risk Factors in Atrial Fibrillation (ATRIA) Study. JAMA. 2001;285(18):2370–2375. 11343485 10.1001/jama.285.18.2370

[38] Miyasaka Y, Barnes ME, Gersh BJ, Cha SS, Bailey KR, Abhayaratna WP, et al Secular trends in incidence of atrial fibrillation in Olmsted County, Minnesota, 1980 to 2000, and implications on the projections for future prevalence. Circulation. 2006;114(2):119–125. 16818816 10.1161/CIRCULATIONAHA.105.595140

[39] Risk factors for stroke and efficacy of antithrombotic therapy in atrial fibrillation. Analysis of pooled data from five randomized controlled trials. Arch Intern Med. 1994;154(13):1449–1457. 8018000

[40] Stewart S, Hart CL, Hole DJ, McMurray JJ. A population-based study of the long-term risks associated with atrial fibrillation: 20-year follow-up of the Renfrew/Paisley study. Am J Med. 2002;113(5):359–364. 12401529 10.1016/s0002-9343(02)01236-6

[41] Dorian P, Jung W, Newman D, Paquette M, Wood K, Ayers GM, et al The impairment of health-related quality of life in patients with intermittent atrial fibrillation: implications for the assessment of investigational therapy. J Am Coll Cardiol. 2000;36(4):1303–1309. 11028487 10.1016/s0735-1097(00)00886-x

[42] Hagens VE, Ranchor AV, Van Sonderen E, Bosker HA, Kamp O, Tijssen JG, et al Effect of rate or rhythm control on quality of life in persistent atrial fibrillation. Results from the Rate Control Versus Electrical Cardioversion (RACE) Study. J Am Coll Cardiol. 2004;43(2):241–247. 14736444 10.1016/j.jacc.2003.08.037

[43] Hohnloser SH, Kuck KH, Lilienthal J. Rhythm or rate control in atrial fibrillation—Pharmacological Intervention in Atrial Fibrillation (PIAF): a randomised trial. Lancet. 2000;356(9244):1789–1794. 11117910 10.1016/s0140-6736(00)03230-x

[44] Hakalahti A, Biancari F, Nielsen JC, Raatikainen MJ. Radiofrequency ablation vs. antiarrhythmic drug therapy as first line treatment of symptomatic atrial fibrillation: systematic review and meta-analysis. Europace. 2015;17(3):370–378. 10.1093/europace/euu376 25643988

[45] Verma A, Jiang CY, Betts TR, Chen J, Deisenhofer I, Mantovan R, et al Approaches to catheter ablation for persistent atrial fibrillation. N Engl J Med. 2015;372(19):1812–1822. 10.1056/NEJMoa1408288 25946280

[46] Brooks AG, Stiles MK, Laborderie J, Lau DH, Kuklik P, Shipp NJ, et al Outcomes of long-standing persistent atrial fibrillation ablation: a systematic review. Heart Rhythm. 2010;7(6):835–846. 10.1016/j.hrthm.2010.01.017 20206320

