# Supplementary material for: Radiofrequency Catheter Ablation Improves the Quality of Life Measured with a Short Form-36 Questionnaire in Atrial Fibrillation Patients: A Systematic Review and Meta-Analysis
Source: PLoS One. 2016 Sep 28;11(9):e0163755. doi: 10.1371/journal.pone.0163755 (PMC5040266; doi:10.1371/journal.pone.0163755)
Supplement: S1 Fig — PRISMA: Preferred Reporting Items for Systematic reviews and Meta-Analyses. (DOCX) [file pone.0163755.s001.docx]

**
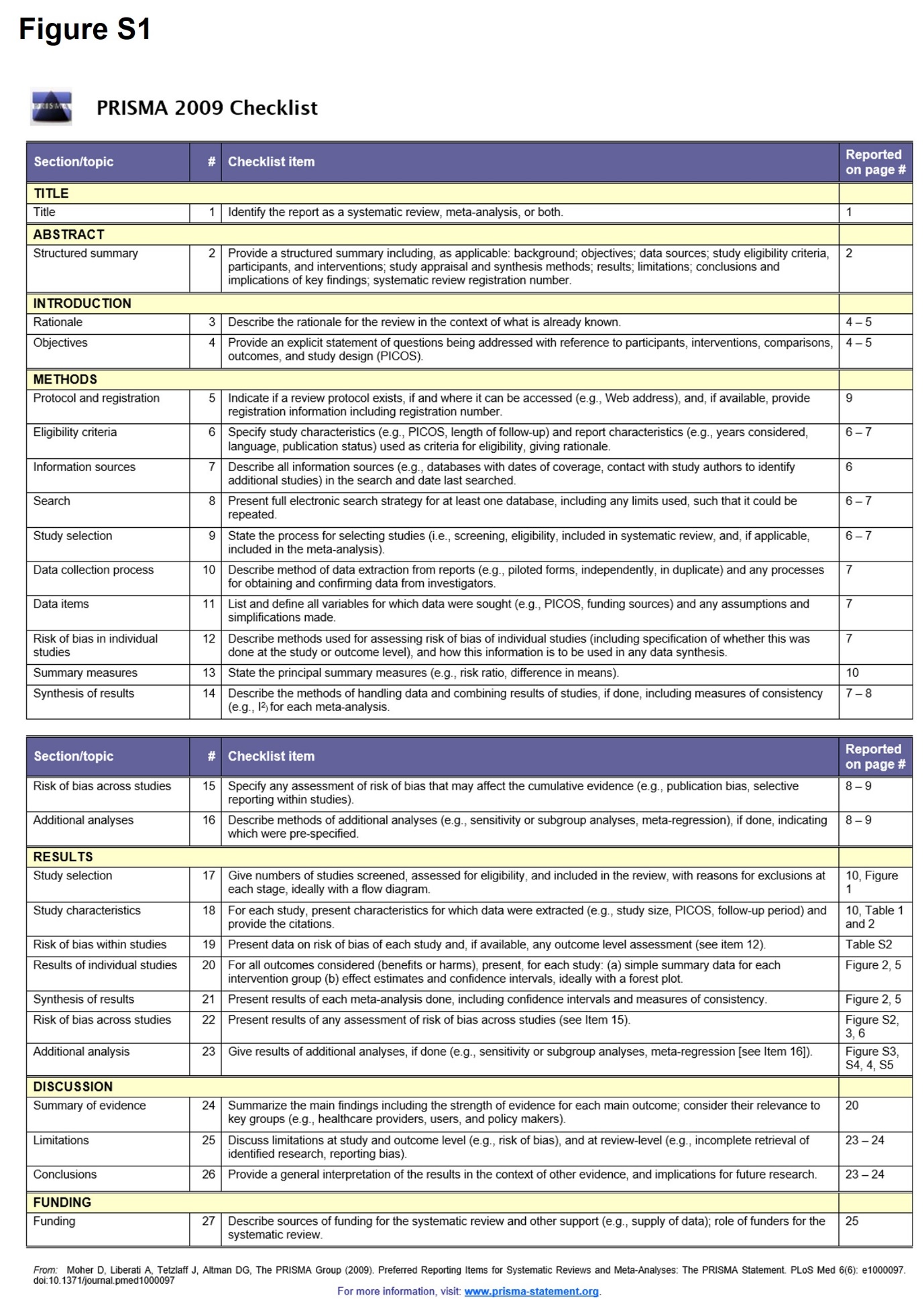
**

**S1 Fig. Checklist of the PRISMA guidelines.**

PRISMA: Preferred Reporting Items for Systematic reviews and Meta-Analyses.
