# Supplementary material for: Radiofrequency Catheter Ablation Improves the Quality of Life Measured with a Short Form-36 Questionnaire in Atrial Fibrillation Patients: A Systematic Review and Meta-Analysis
Source: PLoS One. 2016 Sep 28;11(9):e0163755. doi: 10.1371/journal.pone.0163755 (PMC5040266; doi:10.1371/journal.pone.0163755)
Supplement: S2 Fig — Publication bias was suspected in both the PCS (A) and MCS (B). MCS: mental component summary score; PCS: physical component summary score; RFCA: radiofrequency catheter ablation; WMD: weighted mean difference. (DOCX) [file pone.0163755.s002.docx]

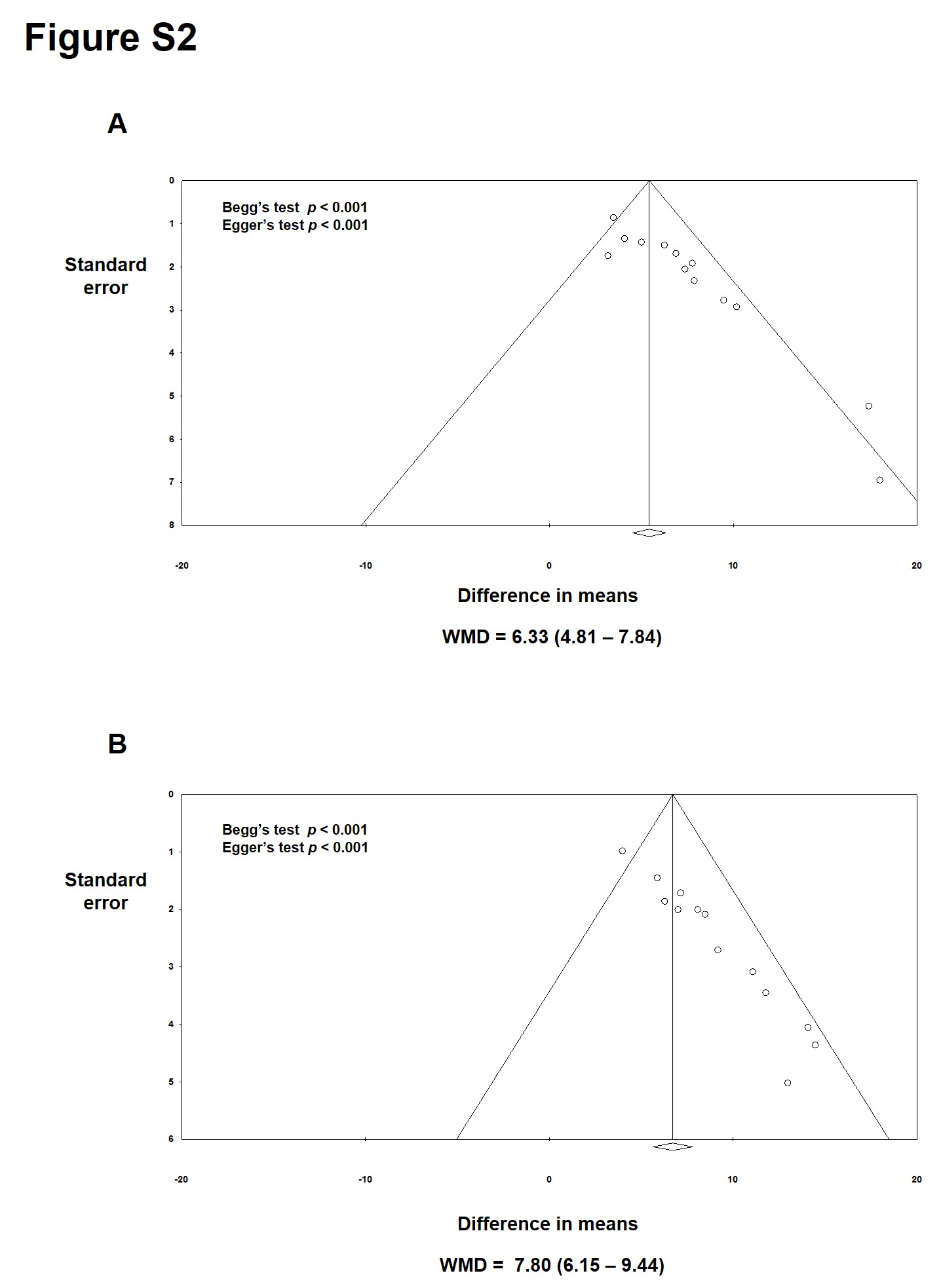


**S2 Fig. Funnel plot analysis: pre-RFCA vs. post-RFCA.**

Publication bias was suspected in both the PCS **(A)** and MCS **(B)**.

MCS: mental component summary score; PCS: physical component summary score; RFCA: radiofrequency catheter ablation; WMD: weighted mean difference.
