# Supplementary material for: Radiofrequency Catheter Ablation Improves the Quality of Life Measured with a Short Form-36 Questionnaire in Atrial Fibrillation Patients: A Systematic Review and Meta-Analysis
Source: PLoS One. 2016 Sep 28;11(9):e0163755. doi: 10.1371/journal.pone.0163755 (PMC5040266; doi:10.1371/journal.pone.0163755)
Supplement: S5 Fig — (A) There was no significant difference in the pooled WMD of the PCS and MCS between studies including only paroxysmal AF vs. studies including various types of AF. (B) Performing additional procedures such as linear ablation or CFAE ablation was not associated with improved outcomes compared to performing pulmonary vein isolation only. AF: atrial fibrillation; CFAE: complex fractionated atrial electrogram; MCS: mental component summary score; PCS: physical component summary score; RFCA: radiofrequency catheter ablation; WMD: weighted mean difference. (DOCX) [file pone.0163755.s005.docx]

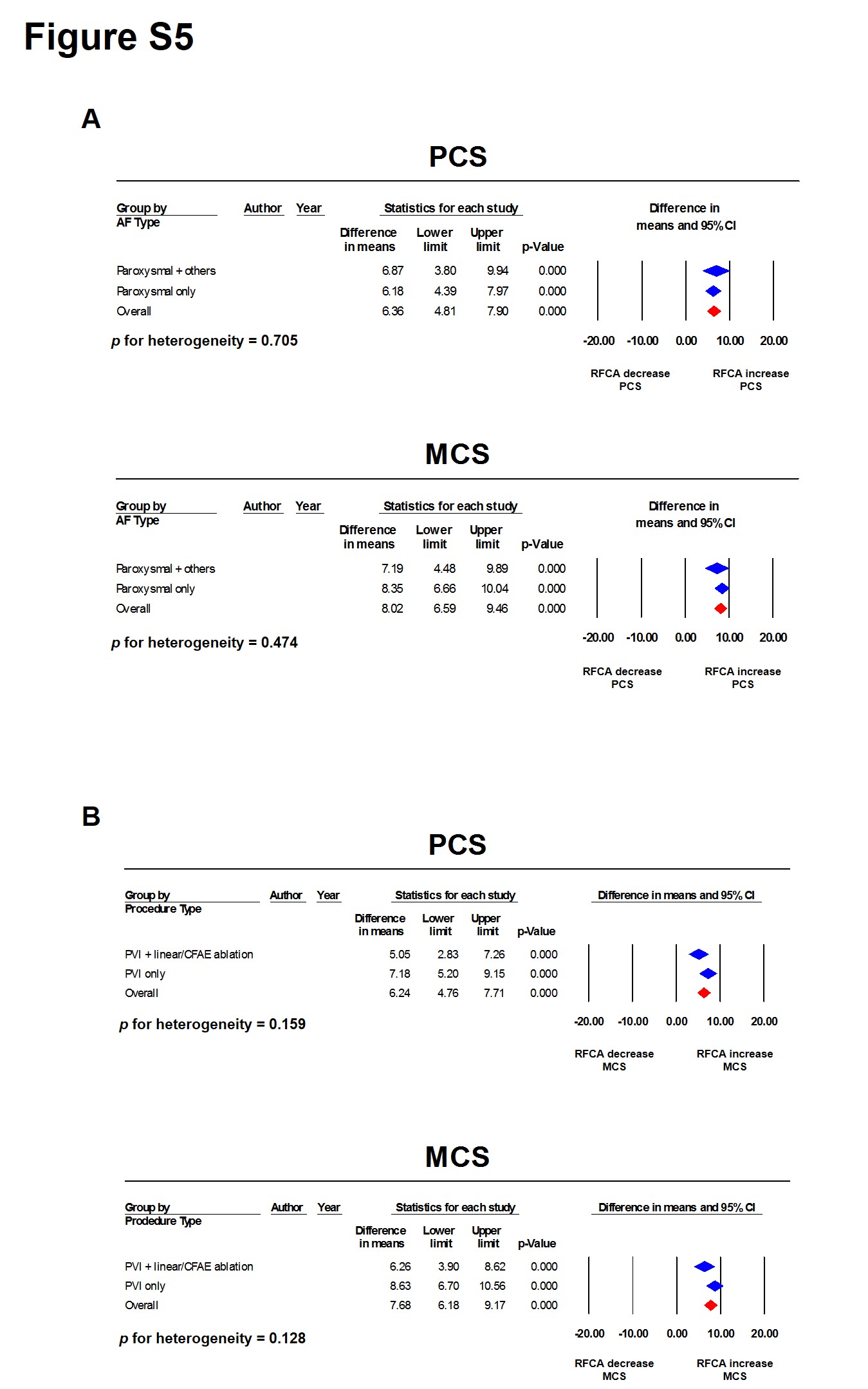


**S5 Fig. Subgroup analysis according to the composition of AF type and procedure type.**

**(A)** There was no significant difference in the pooled WMD of the PCS and MCS between studies including only paroxysmal AF vs. studies including various types of AF.

**(B)** Performing additional procedures such as linear ablation or CFAE ablation was not associated with improved outcomes compared to performing pulmonary vein isolation only.

AF: atrial fibrillation; CFAE: complex fractionated atrial electrogram; MCS: mental component summary score; PCS: physical component summary score; RFCA: radiofrequency catheter ablation; WMD: weighted mean difference.
