# Supplementary material for: Radiofrequency Catheter Ablation Improves the Quality of Life Measured with a Short Form-36 Questionnaire in Atrial Fibrillation Patients: A Systematic Review and Meta-Analysis
Source: PLoS One. 2016 Sep 28;11(9):e0163755. doi: 10.1371/journal.pone.0163755 (PMC5040266; doi:10.1371/journal.pone.0163755)
Supplement: S1 Table — (DOCX) [file pone.0163755.s007.docx]

**S1 Table. Search strategy.**

|  | | Pubmed | Scopus |
| --- | --- | --- | --- |
| Final search | #4 and #8 and #9 | 51 | 411 |
| #9 | ‘ablation’ | 70897 | 361797 |
| #8 | #5 or #6 or #7 | 20677 | 127583 |
| #7 | ‘short form 36’ | 13046 | 107780 |
| #6 | ‘short form-36’ | 7328 | 22015 |
| #5 | ‘SF-36’ | 14890 | 56134 |
| #4 | #1 or #2 or #3 | 68980 | 279381 |
| #3 | ‘AF’ | 27994 | 174392 |
| #2 | ‘a-fib’ | 88 | 857 |
| #1 | ‘atrial fibrillation’ | 56235 | 124994 |

AF and A-fib: atrial fibrillation; SF-36: short form-36.
