# Supplementary material for: Radiofrequency Catheter Ablation Improves the Quality of Life Measured with a Short Form-36 Questionnaire in Atrial Fibrillation Patients: A Systematic Review and Meta-Analysis
Source: PLoS One. 2016 Sep 28;11(9):e0163755. doi: 10.1371/journal.pone.0163755 (PMC5040266; doi:10.1371/journal.pone.0163755)
Supplement: S2 Table — (DOCX) [file pone.0163755.s008.docx]

**S2 Table. The Newcastle-Ottawa scale for assessing the quality of 16 non-randomized studies included in the meta-analysis.**

**(A)** The Newcastle-Ottawa scoring system (downloaded from the homepage of Ottawa hospital. http://www.ohri.ca/programs/clinical_epidemiology/oxford.asp)


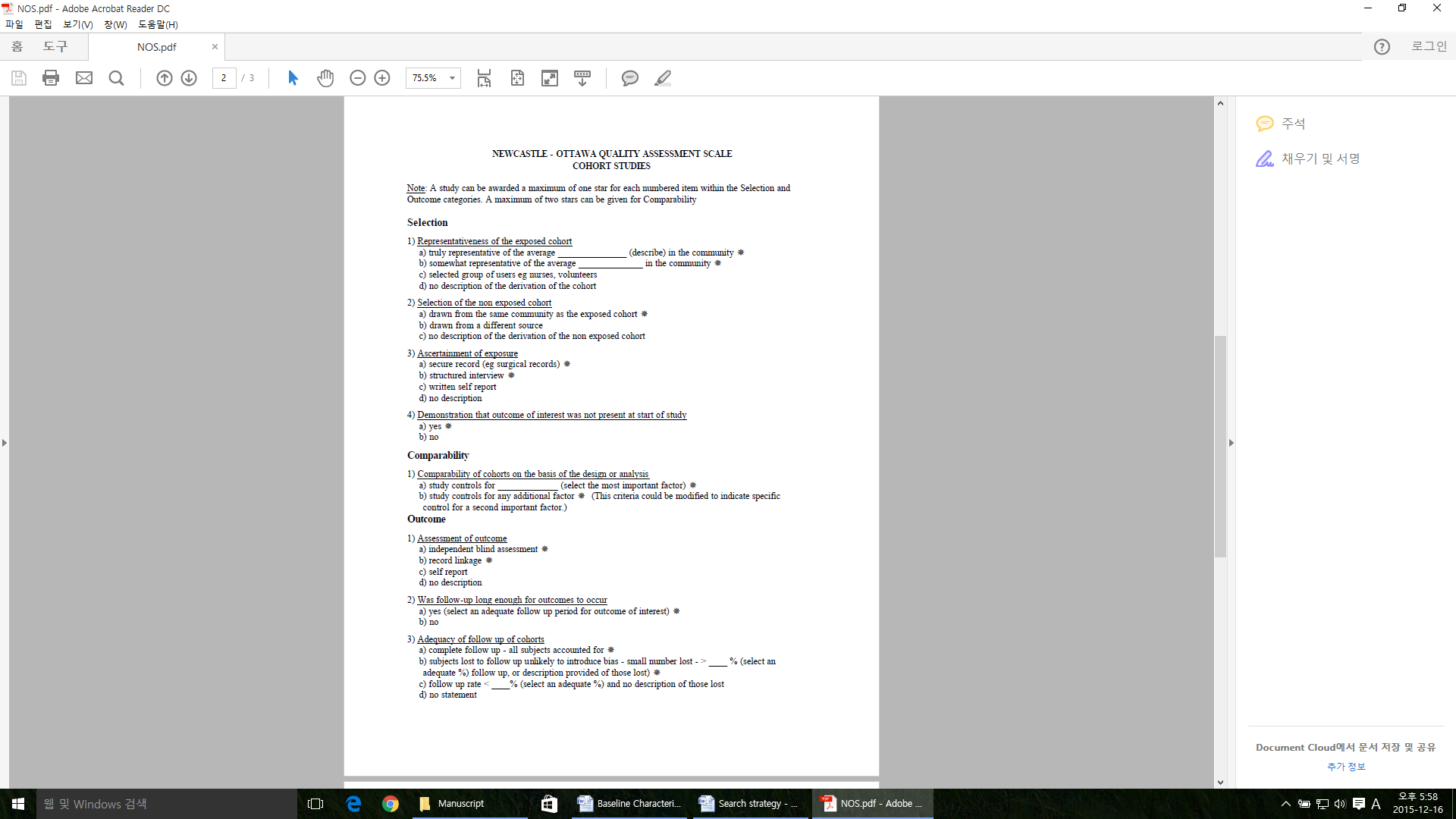


**(B)** pre-RFCA vs. post-RFCA

| Author | Year | Selection | Comparability | Outcome |
| --- | --- | --- | --- | --- |
| Tada et al. | 2003 | ★★★ | ★★ | ★★ |
| Pürerfellner et al. | 2004 | ★★★ | ★★ | ★★ |
| Cha et al. | 2008 | ★★★★ | ★★ | ★ |
| Carnlöf et al. | 2010 | ★★★★ | ★★ | ★★ |
| Wokhlu et al. | 2010 | ★★★★ | ★★ | ★★ |
| Reynolds et al. | 2010 | ★★★ | ★★ | ★ |
| Pappone et al. | 2011 | ★★★ | ★★ | ★★ |
| Höglund et al. | 2013 | ★★★★ | ★★ | ★★ |
| Mantovan et al. | 2013 | ★★★★ | ★★ | ★★ |
| Sang et al. | 2013 | ★★★ | ★★ | ★★ |
| Efremidis et al. | 2014 | ★★★ | ★★ | ★★ |
| Natale et al. | 2014 | ★★★ | ★★ | ★★ |
| Wynn et al. | 2015 | ★★★★ | ★★ | ★★ |

**(C)** Treatment success group vs. AF recurrence group

| Author | Year | Selection | Comparability | Outcome |
| --- | --- | --- | --- | --- |
| Wokhlu et al. | 2010 | ★★★★ | ★★ | ★★ |
| Mohanty et al. | 2012 | ★★★★ | ★★ | ★★ |
| Sang et al. | 2013 | ★★★ | ★★ | ★★ |
| Gu et al. | 2013 | ★★★★ | ★★ | ★★ |
| Mohanty et al. | 2014 | ★★★ | ★★ | ★★ |

AF: atrial fibrillation; RFCA: radiofrequency catheter ablation.
