# Supplementary material for: Radiofrequency Catheter Ablation Improves the Quality of Life Measured with a Short Form-36 Questionnaire in Atrial Fibrillation Patients: A Systematic Review and Meta-Analysis
Source: PLoS One. 2016 Sep 28;11(9):e0163755. doi: 10.1371/journal.pone.0163755 (PMC5040266; doi:10.1371/journal.pone.0163755)
Supplement: S3 Table — (DOCX) [file pone.0163755.s009.docx]

**S3 Table. Procedure related complications of each study.**

**(A)** pre-RFCA vs. post-RFCA

| Source (Year) | Complication rate (%) | CVA or TIA | PV stenosis | PV stenosis with Sx | PV stenosis without Sx | Pericardial effusion | Hemothorax | Pericarditis | Heart failure | AV block | Pneumonia | Pulmonary edema | Paralysis of diaphragm | Thyrotoxicosis | Neuropathy | Vascular complications |
| --- | --- | --- | --- | --- | --- | --- | --- | --- | --- | --- | --- | --- | --- | --- | --- | --- |
| Tada et al.  (2003) | 26.0% (13/50) | 0 | 13 | 0 | 13 | 0 | 0 | 0 | 0 | 0 | 0 | 0 | 0 | 0 | 0 | 0 |
| Pürerfellner et al.  (2004) | 14.7% (11/75) | 0 | 6 | 3 | 3 | 1 | 1 | 1 | 0 | 0 | 0 | 0 | 0 | 1 | 1 | 0 |
| Cha et al.  (2008) | 5.2 % (27/523) | 4 | 7 | NR | NR | 12 | 0 | 0 | 0 | 0 | 0 | 0 | 4 | 0 | 0 | 0 |
| Carnlöf et al.  (2010) | NR | NR | NR | NR | NR | NR | NR | NR | NR | NR | NR | NR | NR | NR | NR | NR |
| Wokhlu et al.  (2010) | 8.6% (43/502) | 9 | 24 | 4 | 20 | 10 | 0 | 0 | 0 | 0 | 0 | 0 | 0 | 0 | 0 | 0 |
| Reynolds et al.  (2010) | 4.9% (5/103) | 0 | 0 | 0 | 0 | 1 | 0 | 0 | 1 | 0 | 1 | 1 | 0 | 0 | 0 | 1 |
| Pappone et al.  (2011) | 5.1% (5/99) | 1 | 0 | 0 | 0 | 1 | 0 | 0 | 0 | 0 | 0 | 0 | 0 | 0 | 0 | 3 |
| Höglund et al.  (2013) | NR | NR | NR | NR | NR | NR | NR | NR | NR | NR | NR | NR | NR | NR | NR | NR |
| Mantovan et al.  (2013) | 8.0% (8/100) | 0 | 1 | 0 | 1 | 2 | 0 | 0 | 0 | 0 | 0 | 0 | 0 | 0 | 0 | 5* |
| Sang et al.  (2013) | 0.0% (0/82) | 0 | 0 | 0 | 0 | 0 | 0 | 0 | 0 | 0 | 0 | 0 | 0 | 0 | 0 | 0 |
| Efremidis et al.  (2014) | NR | NR | NR | NR | NR | NR | NR | NR | NR | NR | NR | NR | NR | NR | NR | NR |
| Natale et al.  (2014) | 7.5% (12/161) | 0 | 0 | 0 | 0 | 4 | 0 | 3 | 0 | 1 | 0 | 0 | 0 | 0 | 0 | 4 |
| Wynn et al.  (2015) | 8.1% (10/124) | 0 | 0 | 0 | 0 | 1 | 0 | 0 | 2 | 0 | 0 | 0 | 0 | 0 | 0 | 7 |

**(B)** Treatment success group vs. AF recurrence group

| Source (Year) | Complication rate (%) | CVA or TIA | PV stenosis | PV stenosis with Sx | PV stenosis without Sx | Pericardial effusion | Hemothorax | Pericarditis | Heart failure | AV block | Pneumonia | Pulmonary edema | Paralysis of diaphragm | Thyrotoxicosis | Neuropathy | Vascular complications |
| --- | --- | --- | --- | --- | --- | --- | --- | --- | --- | --- | --- | --- | --- | --- | --- | --- |
| Wokhlu et al.  (2010) | 8.6% (43/502) | 9 | 24 | 4 | 20 | 10 | 0 | 0 | 0 | 0 | 0 | 0 | 0 | 0 | 0 | 0 |
| Mohanty et al. (2012) | 0.9% (14/1496) | 0 | 0 | 0 | 0 | 11 | 0 | 0 | 0 | 0 | 0 | 0 | 0 | 0 | 0 | 3 |
| Sang et al.  (2013) | 0.0% (0/82) | 0 | 0 | 0 | 0 | 0 | 0 | 0 | 0 | 0 | 0 | 0 | 0 | 0 | 0 | 0 |
| Gu et al.  (2013) | 2.7% (15/550) | 1 | 0 | 0 | 0 | 8 | 0 | 0 | 0 | 0 | 0 | 0 | 0 | 0 | 0 | 6 |
| Mohanty et al.  (2014) | 1.6% (1/61) | 0 | 0 | 0 | 0 | 1 | 0 | 0 | 0 | 0 | 0 | 0 | 0 | 0 | 0 | 0 |

* Includes minor bleeing

AV: atrioventricular; CVA: cerebrovascular accident; NR: not reported; PV: pulmonary vein; Sx: symptoms; TIA: transient ischemic attack.
