# Supplementary material for: Radiofrequency Catheter Ablation Improves the Quality of Life Measured with a Short Form-36 Questionnaire in Atrial Fibrillation Patients: A Systematic Review and Meta-Analysis
Source: PLoS One. 2016 Sep 28;11(9):e0163755. doi: 10.1371/journal.pone.0163755 (PMC5040266; doi:10.1371/journal.pone.0163755)
Supplement: S4 Table — (DOCX) [file pone.0163755.s010.docx]

**S4 Table. Anticoagulation therapy before and after RFCA.**

**(A)** pre-RFCA vs. post-RFCA

| Source (Year) | Anticoagulation after RFCA | Duration after RFCA | Anticoagulation before RFCA | Duration before RFCA |
| --- | --- | --- | --- | --- |
| Tada et al.  (2003) | NR | NR | NR | NR |
| Pürerfellner et al.  (2004) | NR | NR | NR | NR |
| Cha et al.  (2008) | NR | NR | NR | NR |
| Carnlöf et al.  (2010) | NR | NR | Warfarin | NR |
| Wokhlu et al.  (2010) | Warfarin | > 3 months | Warfarin (64%) | NR |
| Reynolds et al.  (2010) | Warfarin | > 3 months | NR | NR |
| Pappone et al.  (2011) | Warfarin | Variable duration based on CHADS2 score | Warfarin | NR |
| Höglund et al.  (2013) | NR | NR | NR | NR |
| Mantovan et al.  (2013) | Warfarin | > 3 months | Warfarin | > 4 weeks |
| Sang et al.  (2013) | Warfarin | Discontinued after 3 months if no AF was observed | NR | NR |
| Efremidis et al.  (2014) | Warfarin | > 3 months | Warfarin | NR |
| Natale et al.  (2014) | Warfarin | > 3 months | NR | NR |
| Wynn et al.  (2015) | Warfarin or dabigatran | NR | Warfarin or dabigatran | NR |

**(B)** Treatment success group vs. AF recurrence group

| Source (Year) | Anticoagulation after RFCA | Duration after RFCA | Anticoagulation before RFCA | Duration before RFCA |
| --- | --- | --- | --- | --- |
| Wokhlu et al.  (2010) | Warfarin | > 3 months | Warfarin (64%) | NR |
| Mohanty et al.  (2012) | Warfarin | NR | Warfarin | NR |
| Sang et al.  (2013) | Warfarin | Discontinued after 3 months if no AF was observed | NR | NR |
| Gu et al.  (2013) | Warfarin | NR | Warfarin | > 1 month |
| Mohanty et al.  (2014) | NR | NR | NR | NR |

AF: atrial fibrillation; NR: not reported; RFCA: radiofrequency catheter ablation.
